# Supplementary material for: Machine learning and causal inference applied to the gut metagenome-metabolome axis reveals a link between neonatal jaundice and autism spectrum disorder
Source: mSystems. 2026 Jan 9;11(2):e01405-25. doi: 10.1128/msystems.01405-25 (PMC12911356; doi:10.1128/msystems.01405-25)
Supplement: Supplemental Material — Supplemental figures and methods. [file msystems.01405-25-s0001.docx]

**Supplementary Appendix**

**Supplementary Figures**

**
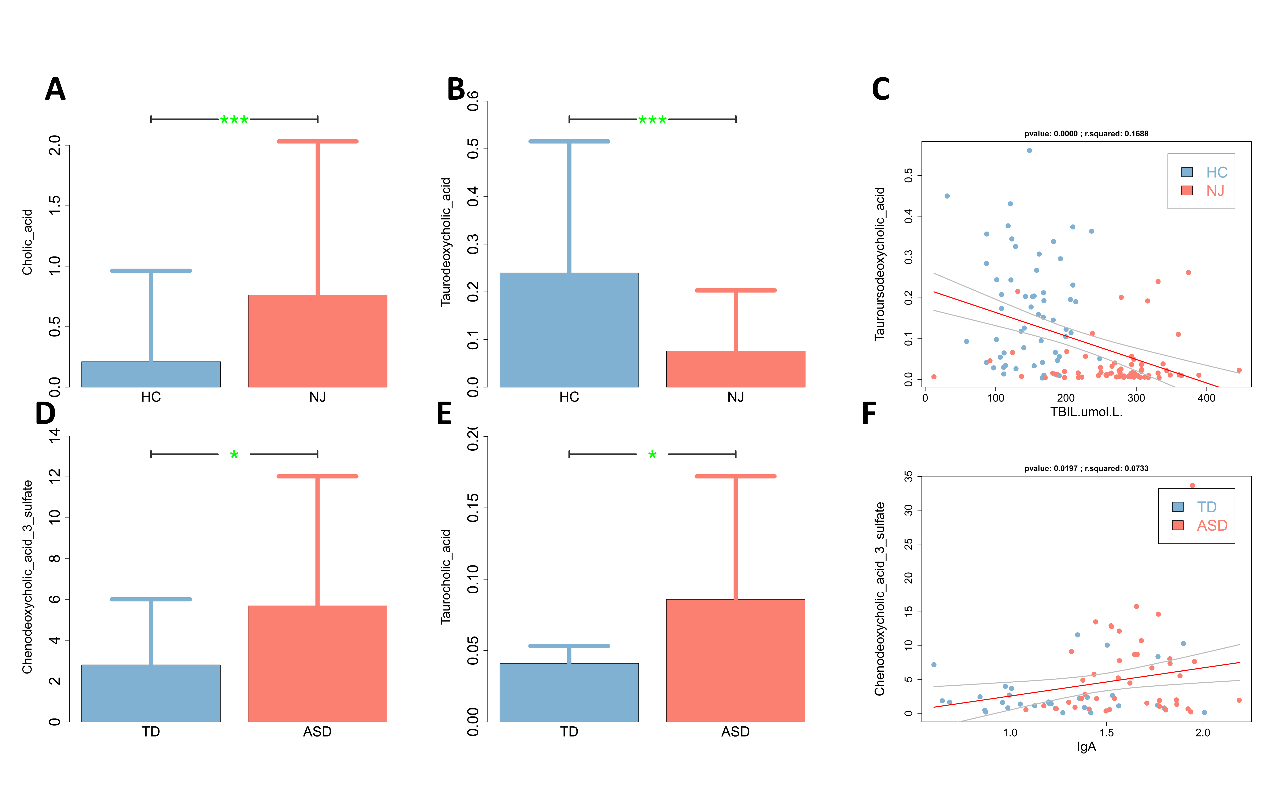
**

**Supplementary** **Figure 1. Both children with neonatal jaundice (NJ) and autism spectrum disorder (ASD) display changes in intestinal bile acid metabolism**. **A–C**. Alterations of gut bile acid metabolism in patients with NJ. We found that gut bile acid (cholic acid) content was significantly higher in patients with NJ than in healthy controls (HCs; **A**), and tauroursodeoxycholic acid content was significantly lower in the NJ group than in the HC group (**B**). Meanwhile, tauroursodeoxycholic acid levels were negatively correlated with total serum bilirubin levels (**C**). **D–F**. Alterations of gut bile acid metabolism in patients with ASD. We found that the levels of the gut bile acid metabolites chenodeoxycholic acid-3-sulfate (**D**) and taurocholic acid (**E)** levels were significantly higher in children with ASD than in typically developing (TD) children, and gut chenodeoxycholic acid-3-sulfate levels positively correlated with IgA levels (**F**).


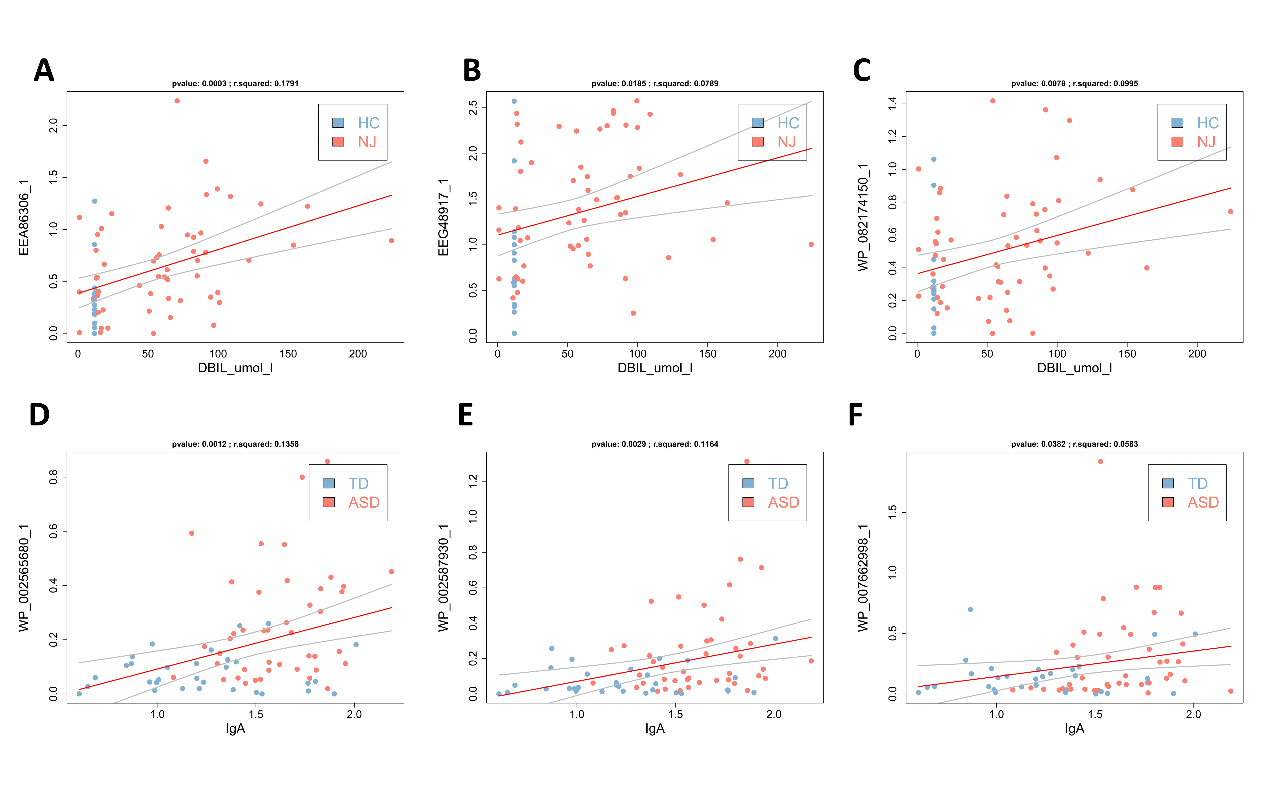


**Supplementary** **Figure 2. The abundance of gut bile acid metabolism genes is correlated with clinical** **indices.** **A–C**. The abundance of gut bile acid metabolism genes is positively correlated with serum direct bilirubin (DBIL) levels in children with neonatal jaundice (NJ). **A**. DBIL_umol_l-VS-EEA86306_1. **B**. DBIL_umol_l-VS-EEG48917_1. **C**, DBIL_umol_l-VS-WP_ 082174150_1. **D–F.** The abundance of gut bile acid metabolism genes is positively correlated with IgA content in children with autism spectrum disorder (ASD). **D**. IgA-VS-WP_002565680_1. **E**. IgA-VS-WP_002587930_1. **F**. IgA-VS-WP_007662998_1. HC, healthy control; TD, typically developing.


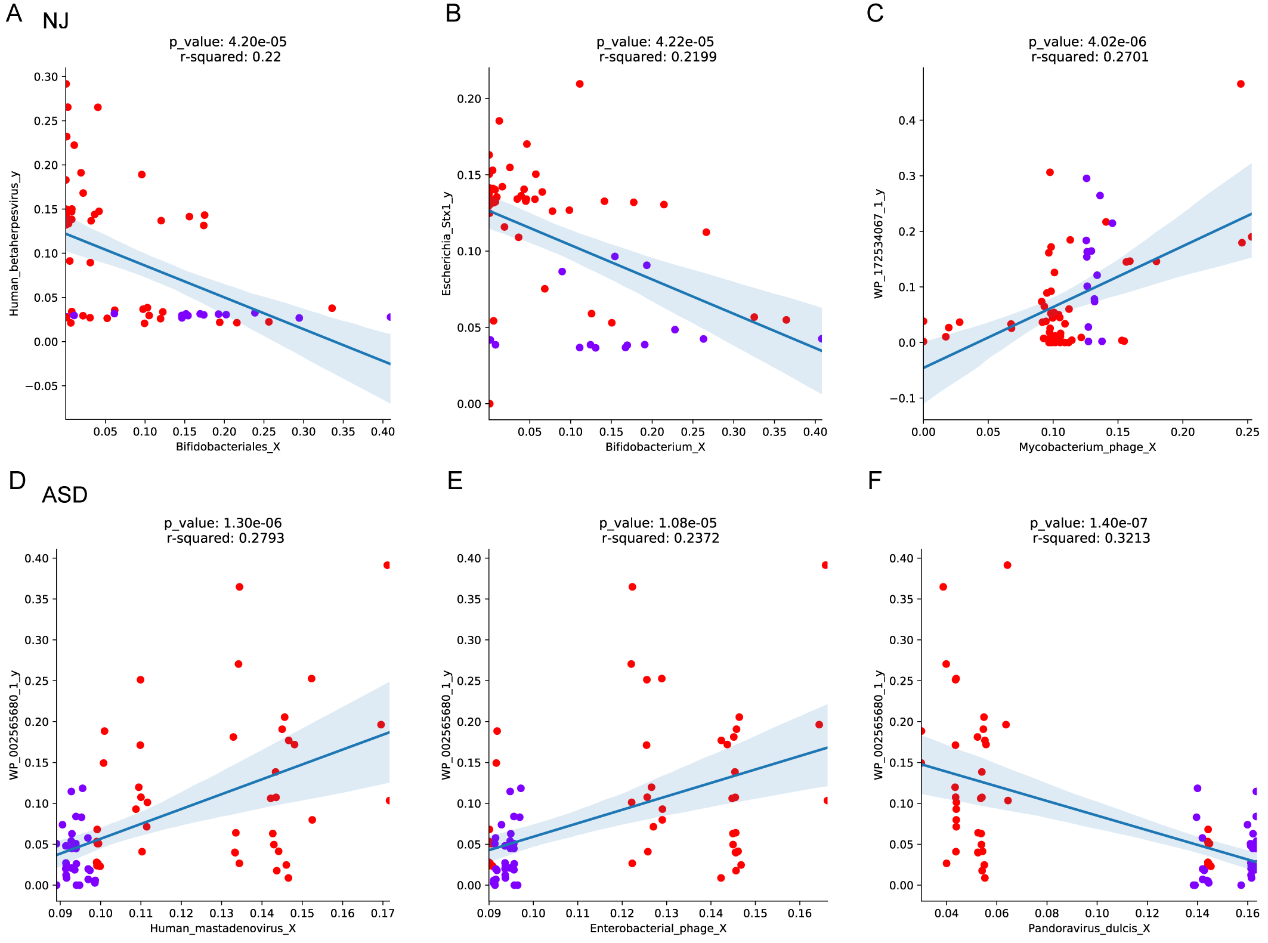


**Supplementary Figure 3.** **Visualization of significant correlations between microorganisms and viruses. A-C.** Correlation between intestinal microbes and viruses associated with NJ. **A.** *Bifidobacteriales* was negatively correlated with *Human betaherpesvirus* (p=4.20e-05, r-squared=0.22). **B.** *Bifidobacterium* was negatively correlated with *Escherichia Stx1* (p=4.22e-05, r-squared=0.2199). **C.** *Mycobacterium phage* was positively correlated with WP 17534087 (p=4.02e-06, r-squared=0.2701). **D-F.** Correlation between intestinal microbes and viruses associated with ASD. **D.** *Human mastadenovirus* was positively correlated with WP 002565860_1 (p=1.30e-06, r-squared=0.2793). **E.** *Enterobacterial phage* was negatively correlated with WP 002565860_1 (p=1.08e-05, r-squared=0.2372). **F.** *Pandoravirus dulcis* was negatively correlated with WP 002565860_1 (p=1.40e-07, r-squared=0.3213).

**Supplementary methods**

***Participant recruitment and sample collection*: NJ cohort1**: includes 68 cases of neonatal jaundice (NJ) and 68 matched healthy controls (HC), neonatal jaundice (NJ), also known as neonatal hyperbilirubinemia, was diagnosed according to the American Academy of Pediatrics Guidelines for Neonatal Jaundice Intervention (PMID: 15231951) and the Expert Consensus on the Diagnosis and Treatment of Neonatal Hyperbilirubinemia by the Neonatology Group of the Chinese Medical Association's Pediatrics Branch (PMID: 25537539). All samples were obtained from the Shenzhen Longhua District People's Hospital. The inclusion and exclusion criteria are detailed in a previous article (submitted) and briefly described as follows. Inclusion criteria: no prenatal high-risk factors, no antibiotics, no fetal defecation. Exclusion criteria: neonates with high-risk factors, antibiotic use within 2 weeks prenatally, younger than gestational age, with severe infection or presence of congenital malformations. The first meconium excreted by the newborns included in the study after birth, in a volume of 3-5 g, was placed in a -80 ℃ refrigerator for backup. Participants were eventually classified as NJ and HC based on serum bilirubin levels during the child's hospitalization. **NJ cohort2**: the NJ cohort2 included 56 infants with NJ and 14 infants with HC, with participant information detailed in our previously study (PMID: 30342317) and briefly described as follows: all participants were from Shenzhen Children's Hospital, of all the infants with NJ, 40 had neonatal cholestasis (NC), diagnosed according to the North American Society for Pediatric Gastroenterology, Hepatology and Nutrition guidelines for the evaluation of cholestatic jaundice in infants (PMID: 27429428). Sixteen infants of breast milk jaundice (BMJ) were diagnosed as follows: Breastfed newborns with jaundice lasting longer than physiological jaundice or disappearing after cessation of breastfeeding, in good general condition, without hepatomegaly, and with normal liver function. HC group: Healthy control infants were <6 months old, full-term gestational age, non-jaundiced infants. Exclusion criteria: >6 months of age, premature birth, antibiotic-using infants before 2 weeks of age. **ASD cohort**: 43 children with ASD and 31 children with typical developing (TD) were included. The participant information is detailed in previously studies (PMID: 30701194, 30394313, 33510860), briefly described as follows: ASD was diagnosed based on the guidelines in the Diagnostic and Statistical Manual of Mental Disorders, 5th edition, for children aged 2-8 years with a diagnosis of ASD "requiring very high support". The controls were sex- and age-matched TD children, normal children attending kindergarten/primary school entrance physical examinations, and all participants were from Shenzhen Children's Hospital.

Participants' guardians collected stool samples in sterile containers and transported them overnight on ice to the laboratory, where researchers immediately dispensed them according to 3-5 g/tube and stored them in the -80°C refrigerator. The study protocol was approved by the hospital medical ethics committee, and each child's parents provided written informed consent. The study protocol was in accordance with the Declaration of Helsinki and was approved by the hospital medical ethics committee.

***16S rRNA gene sequencing and bioinformatics*** we completed 16S rRNA gene sequencing and bioinformatics analysis, which are briefly described as follows. **16S rRNA gene sequencing**: first, extracted fecal DNA was diluted to 1 ng /μl as template DNA, and bacterial 16S rRNA gene V4 region universal primers 515F (5'-GTG CCA GCM GCC GCG GTA A-3') and 806R (5'-GGA CTA CNN GGG TAT CTA AT-3') were used to perform polymerase chain reaction (PCR), and the PCR was performed using the Phusion® High Fidelity PCR Kit (New England Biolabs, Ipswich, MA, USA); then, the QIAquick Gel Extraction Kit (Qiagen, Hilden, Germany) was used to recover the target PCR products; then, the TruSeq® DNA PCR-Free Sample Preparation Kit (Illumina, San Diego, California, USA) was used to construct the library, and finally, HiSeq2500 (Illumina, San Diego, California, USA) was used to sequence the library in 250 bp paired-end mode. **16S rRNA gene bioinformatic analysis**: firstly, double-end reads were merged using FLASH software (version 1.2.7), then QIIME 2 software (version 2020.11) was used for quality control to obtain clean tags (clean tags) and aligned clean tags to the Gold database (version 20110519), then UCHIME software (version 7.0.1001) was used to detect chimeric sequences, Uparse software (version 7.0.1001) was used to cluster non-chimeric clean tags into operational taxonomic units (OTUs) according to a similarity threshold of ≥97%; finally mothur software was used for OTU alignment with the SILVA database and species taxonomic annotation, and MUSCLE software (version 3.8.31) was used to investigate OTUs in phylogenetic relationship with the SILVA database, and the number of OTU reads was normalized according to the minimum amount of data in the sample before statistical analysis. **Differential analysis of gut microbiota:** In order to screen for taxonomies that differed significantly between groups, species with low abundance (mean relative abundance less than 0.1%) and low coverage (no taxonomy detected in more than 20% of samples) were first filtered out. the DESeq2 method was used to find taxonomies that differed significantly between groups, where the thresholds set were: p-value less than 0.05, Benjamini and Hochberg false discovery rate (FDR) calibrated p-value less than 0.001, and |log2foldchange| >1.2.

***LC-MS/MS metabolome*** First, the Acquity Ultra Performance LC-QTOF system (Waters Corporation, Milford, MA, USA) was used to separate the metabolites by liquid chromatography, then time-of-flight quantitative mass spectrometry (Waters Corporation) was used to further detect the molecular weight of the metabolites; finally, Masslynx 4.1 software (Waters Corporation) was used to Waters Corporation) was used to pre-process the acquired metabolite composition data to obtain a two-dimensional data matrix containing retention time (RT), positive or negative mode mass-to-charge ratio (MZ) and peak intensity. **Differential analysis of gut Metabolites**. To discover metabolite compositions that differed significantly between groups, first, metabolites with low abundance (mean relative abundance <0.001%) and low coverage (no metabolites detected in more than 20% of samples) were filtered out, and then the DESeq2 method was used to discover metabolites that differed significantly between groups, where the thresholds set were: p-value <0.05, Benjamini and Hochberg false discovery rate (FDR) calibrated to a P-value less than 0.001, and |log2foldchange| >0.58.

**Gut Metagenomic analysis** Referring to our previous studies (PMID: 27583441, 30394313, 30701194, 34504805), we completed the metagenome sequencing, which is briefly described as follows: First, KneadData software (version 0.7.4) (http://huttenhower.sph.harvard.edu/kneaddata), a tool developed by Huttenhower lab specifically for quality control of metagenome data, was used to complete the quality control and host removal process, this software uses Trimmomatic (version 0.39) for data quality control with the parameter "SLIDINGWINDOW:4:20 MINLEN:50", and use Bowtie2 software (version 2.2.5) to align the sequences to the human genome (version hg38), here with the parameter "--very-sensitive --dovetail", to obtain metagenomic data without human sequence. Then, BLASTX and BLASTN methods of BLAST software (PMID: 2231712) were used to align remaining metagenomic sequence to the National Center for Biotechnology Information (NCBI) Taxonomy database, NCBI Nucleic Acid Database (NCBI-NT) and NCBI Protein Database (NCBI-NR), and finally, MEGAN software ( PMID: 24060133, version 5.0) was used to obtain the gut microbiota composition, including the composition of the gut microbiota at various taxonomic levels such as kingdom, phylum, order, family, genus and species, as well as the functional composition of the gut microbiota based on MEGAN’s SEED classification and Kyoto Encyclopedia of Genes and Genomes (KEGG) classification. **Differential analysis of gut bacteria:** In order to discover gut microbiota composition (taxonomy) that differed significantly between groups, gut bacteria with low abundance (mean relative abundance <0.01%) and low coverage (no bacteria detected in more than 20% of samples) were first filtered out. the DESeq2 method was used to discover gut bacteria that differed significantly between groups, where thresholds were set: p-value <0.05, and |log2foldchange| >0.58. Benjamini and Hochberg false discovery rate (FDR) calibrated p-values less than 0.05, and |log2foldchange| >0.58.

**Gut bile acid metabolizing genes** To systematically identify gut bacteria containing bile salt hydrolase (BSH) and hydroxysteroid dehydrogenase (HSDH) genes, First, based on the published articles on BSH and HSDH (PMID: 33938389) and in combination with the UniGene non-redundant gene database from NCBI and the Uniprot protein database (https://www.uniprot.org), the complete bile salt hydrolase (BSH) and hydroxysteroid dehydrogenase (HSDH) gene sets were obtained by manual search, and then all amino acid sequences of bile salt hydrolase (BSH) and hydroxysteroid dehydrogenase (HSDH) were downloaded and combined into a reference sequence file in fasta format. Then, the makedb module of Diamond software was used to build the reference index library; finally, the blastx module of Diamond software was used to align the metagenome sequencing data to the reference index library, with the parameters set were "--evalue 1e-5 -- threads 4 --max-target-seqs 1 --outfmt 6" to obtain the bile salt hydrolase (BSH) and hydroxysteroid dehydrogenase (HSDH) gene abundance files for each sample. **Differential analysis of gut BSH_HSDH genes**: To discover bile acid metabolizing enzyme genes (BSH_HSDH) that differed significantly between groups, BSH_HSDH with low abundance (mean relative abundance <0.01%) and low coverage (no BSH_HSDH detected in more than 50% of samples) were first filtered out. the DESeq2 method was used to discover BSH_HSDH that differed significantly between groups, setting thresholds of P-value less than 0.05, Benjamini and Hochberg false discovery rate (FDR) calibrated p-values less than 0.15, and |log2foldchange| >0.5.

**Gut virome** PathoScope software (version 2.0) (PMID: 23843222, 25225611) is used to identify viral sequences from metagenomic data; PathoScope software is a complete computational framework for strain identification in environmental or clinical sequencing samples; the detailed process can be found in the software manual and is briefly described as follows: First, all viral reference sequences containing complete genomic sequences were downloaded from the NCBI refseq database (October 2019, total 9298 viral complete genome sequences), and Bowtie2 software (PMID: 22388286, version 2.2.5) was used to construct an index file of the reference sequences; then, the MAP module of PathoScope software (version 2.0) was used to compare the metagenomic data with the reference genome; then, the ID module of PathoScope software was used for strain identification; and finally, the REP module of PathoScope software was used to summarize and annotate the results. **Differential analysis of gut virome**: To detect gut virome compositions that differed significantly between groups, low abundance (mean relative abundance <0.0001%) and low coverage (no gut virome composition detected in more than 50% of samples) gut virome compositions were first filtered out. the DESeq2 method was used to detect gut virome compositions that differed significantly between groups, setting thresholds of P-values <0.05, Benjamini and Hochberg false discovery rate (FDR) calibrated to a P-value less than 0.01, and |log2foldchange| >0.2.

**Procrustes Analysis** Procrustes Analysis is a method that compares the consistency of two sets of data by analyzing the shape distribution. Its principle is least-squares orthogonal mapping, that is, through constant iteration to find a canonical shape, and use the least square method to find the affine change of each object shape to this canonical shape. Procrustes Analysis is done through the R package vegan and with reference to the article by Zhao *et al.* (PMID: 30616051). First, the principal component analysis was performed by the rda function to reduce the dimensionality of the two datasets separately, and the coordinates of the feature axes (which represent the linear combination of the variable sets) were extracted for comparison; then the deviation sum of squares M2 statistic and the p-value after 999 permutation tests were obtained by the procrustes function, using the parameters: permutations = how, nperm = 999; finally, the diagram were displayed by the R package ggplot2.

**Causal mediation analysis** The principle of causal mediation analysis is to identify and explain the causal link between the independent variable (X) and the dependent variable (Y) by introducing a mediating variable (M). First, two statistical models were built, namely the model fitY (fitY = lm(Y ~ X + M)) for the dependent variable (Y) to the independent variable (X) and the model fitM (fitM = lm(M ~ X)) for the mediating variable (M) to the independent variable (X). The gvlma() function of the R package gvlma was then used to fit the two models; the mediate() function of the R package mediation (version 4.5.0) was then used to perform the mediation analysis, with parameters set to boot=TRUE, sims=999; by applying a bootstrap with a sampling number of 999, the method obtains the size and p-value of the indirect causal effects (average causal mediation effects, ACME) of the independent variable (X) on the dependent variable (Y) through the mediating variable (M), The size and p-value of the direct effects (average direct effects, ADE) of the independent variable (X) directly on the dependent variable (Y) were also obtained. Finally, the R package ggalluvial was used to visualize the analysis of causal mediation effects.

**Causal inference based on machine learning** Causal inference based on machine learning is done through Microsoft's DoWhy library (https://github.com/microsoft/dowhy) and EconML library (https://github.com/econml/) referencing to the software manual, as detailed in a previous study (accepted), briefly described as follows: The first step is to build a model, that is, to encode our domain knowledge into a causal model and represent it with a graph, where each arrow in the graph indicates a causal relationship: "A->B" means that variable A causes variable B. Here, the outcome is set as a group name here, the outcome is set as a binary variable, where 0 represents the control group and 1 represents the disease group, and the variables of the intervention or treatment are set as clinically important indicators that potentially cause the disease, and other variables to be examined that may be common causes of the intervention or treatment and outcome are set as confounders. The second step, Identification, is based on Dowhy's backdoor.linear_regression method to check whether a given observed variable can estimate the target quantity. The third step, Estimation, is to construct an estimator to compute the estimand identified in the previous step.To model nonlinear data (and data with high-dimensional confounders), we construct the estimator using EconML's machine learning method, which uses gradient boosting trees to learn the relationship between the outcome and confounding factors, as well as the relationship between the intervention and confounding factors, and finally to compare the residuals between the outcome and the intervention. The fourth step, refutation, is to check the robustness of the estimates. This is the most important step in causal inference analysis, and given the absence of a suitable validation set, this step can rely on refutation tests, which use the property of an estimator to refute the correctness of the obtained estimates. Here, we performed a placebo refutation test (placebo_treatment_refuter), which checks whether the estimator returns estimates close to 0 when the intervention variable is replaced by a random variable, and a subsampling refutation test (data_subset_refuter), which collects a random portion of the sample (subset _fraction=0.8) to obtain estimates that are close to those of all samples.
